# Temporal and subgroup disparities in mediation effects on cardiovascular outcomes with liraglutide and semaglutide: a *post-hoc* analysis of LEADER and SUSTAIN-6 trials

**DOI:** 10.1186/s12933-025-03007-w

**Published:** 2025-11-24

**Authors:** Zi-Yang Peng, Yu-Hsuan Lee, Huang-Tz Ou, Shihchen Kuo

**Affiliations:** 1 Institute of Clinical Pharmacy and Pharmaceutical Sciences, College of Medicine, National Cheng Kung University, Tainan, Taiwan; 2 Department of Pharmacy, College of Medicine, National Cheng Kung University, Tainan, Taiwan; 3 Division of Metabolism, Endocrinology and Diabetes, Department of Internal Medicine, University of Michigan Medical School, Ann Arbor, Michigan United States of America

## Abstract

**Background:**

It is unclear whether the time-varying mediation effects of glucagon-like peptide-1 receptor agonists (GLP-1RAs) on the three-point major adverse cardiovascular event (3P-MACE) outcomes, including nonfatal stroke, nonfatal myocardial infarction, and cardiovascular death, via HbA_1c_, urine albumin-to-creatinine ratio (UACR), and systolic blood pressure (SBP) vary by types of GLP-1RAs, and patient subgroups.

**Methods:**

A causal mediation analysis was applied to assess the mediation effects of HbA_1c_, UACR, and SBP on the 3P-MACE with liraglutide and semaglutide over time, using the LEADER and SUSTAIN-6 trials. We further explored the heterogeneity in mediation effects in subsets of vulnerable patients, including those with an estimated glomerular filtration rate (eGFR) level of < 60 ml/min/1.73 m^2^ or established cardiovascular diseases (CVDs).

**Results:**

Individual-level data consisting of 9340 (liraglutide: 4668; placebo: 4672) and 3297 (semaglutide: 1648; placebo: 1649) subjects from the LEADER and SUSTAIN-6 trials, respectively, was utilized for this study. The study population aged around 64.2–64.4/64.4–64.8 years old, with 64.0%–64.5%/58.5%–63.0% of males, and having a diabetes duration of 12.8–12.9/13.2–14.3 years in the LEADER/SUSTAIN-6 trials, respectively. Among study populations, those established CVDs (e.g., heart failure, stroke, myocardial infarction) accounted for 14.0%–35.4% in the LEADER trial, whereas 13.7%–61.9% in the SUSTAIN-6 trial. At the end of each trial, HbA_1c_ contributed the most to liraglutide’s/semaglutide’s effect on 3P-MACE (38.2%, 95% confidence interval: 13.8%–194.5%/51.8%, 29.6%–86.7%), followed by UACR (17.9%, 8.5%–61.9%/12.4%, 6.1%–32.4%) and SBP (6.8%, 2.2%–31.0%/6.7%, 2.9%–11.7%), respectively. Over the trial period, the effect of liraglutide mediated by HbA_1c_ increased from 31.3% to 38.2%, whereas the effects of semaglutide mediated by HbA_1c_ remained stable from 52.5% to 51.8%. For patients with eGFR < 60 ml/min/1.73 m^2^, HbA_1c_ was the leading mediator (35.2%) of semaglutide-associated 3P-MACE, while the mediation effects via HbA_1c_ (7.2%), UACR (11.8%), and SBP (5.1%) on liraglutide-associated 3P-MACE seemed similar and smaller. For patients with established CVDs, HbA_1c_ was the leading mediator of liraglutide- (25.3%) and semaglutide-associated 3P-MACE (51.2%).

**Conclusions:**

The HbA_1c_-mediated effects of liraglutide and semaglutide varied over time and across patient subgroups. HbA_1c_ consistently explained a larger proportion of mediation compared to UACR and SBP, with patterns suggesting that mediation through HbA_1c_ may differ by underlying risk factors.

**Supplementary Information:**

The online version contains supplementary material available at 10.1186/s12933-025-03007-w.

## Background

Given the apparent cardiovascular benefit of glucagon-like peptide-1 receptor agonist (GLP-1RA) therapy in patients with type 2 diabetes (T2D), studies have been conducted to explore the potential mediation mechanisms underlying the additional treatment effect beyond glycemic control, with several clinical biomarkers, including glycated hemoglobin (HbA_1c_), urine albumin-to-creatinine ratio (UACR), and systolic blood pressure (SBP), identified as significant mediators [1, 2]. These possible mechanisms and mediators not only elucidate the new therapeutic era for GLP-1RAs but also suggest optimizing treatment benefits through the effective management of glycemic levels and traditional non-glycemic risk factors for cardiovascular diseases (CVDs) [1–3].

The biomarker mediators of GLP-1RA-associated cardiovascular outcomes were generally determined at a pre-specified time point (e.g., at 3 years following treatment initiation [1, 4]) and their identification mainly relied on trial data for liraglutide [1, 3] and dulaglutide [2]. Although the Semaglutide and Cardiovascular Outcomes in Obesity without Diabetes (SELECT) trial has been applied to explore the mediation mechanisms on cardiovascular outcomes with semaglutide, with HbA_1c_ identified as a significant mediator, this trial predominantly enrolled overweight/obese patients [5], affecting the study’s applicability to general T2D populations. Beyond the static contribution of mediation effects restricted to a certain time point of follow-up, the importance of temporal changes in the mediation effects on GLP-1RA-associated cardiovascular outcomes have been recognized [3]. Understanding the time-varying changes in the mediated impact over time would provide insights for better clinical interpretations and decision-making. However, it remains unclear whether the mediators and their effects on GLP-1RA-associated cardiovascular outcomes differ by type of GLP-1RAs and patient characteristics, especially for vulnerable populations (e.g., those with impaired renal function). Understanding the variation in mediation mechanisms over time and across diverse patient clinical characteristics is thus imperative to support the personalized management of underlying risk factors over the course of T2D treatment.

This study sought to quantify the percentage mediation of liraglutide and semaglutide via HbA_1c_, UACR, and SBP on three-point major adverse cardiovascular event (3P-MACE) outcomes and their mediated effects over time using individual-level data from the Liraglutide and Cardiovascular Outcomes in Type 2 Diabetes (LEADER) and Semaglutide and Cardiovascular Outcomes in Patients with Type 2 Diabetes (SUSTAIN-6) trials. The heterogeneity of mediation effects on GLP-1RA-associated cardiovascular outcomes in subsets of vulnerable patients, including those with an estimated glomerular filtration rate (eGFR) level of < 60 ml/min/1.73 m^2^ and those with established CVDs, was explored.

## Methods

### Data source

The mediation analyses were conducted using all randomized participants from the LEADER trial [6] (liraglutide 4668; placebo 4672) and SUSTAIN-6 trial [7] (semaglutide 1648; placebo 1649). Liraglutide was administered once daily at 1.8 mg (with lower doses permitted during titration), and semaglutide was administered once weekly at 0.5 mg or 1.0 mg, according to the trial protocols. Full inclusion and exclusion criteria are summarized in Supplementary Table 1. Mediation analysis is hypothesis-driven and requires high-quality datasets with systematic and repeated biomarker measurements [8]. Randomized controlled trial data provide this advantage because randomization reduces unmeasured confounding, and trial protocols ensure the completeness and consistent collection of key biomarkers. Previous studies have also demonstrated the utility of RCT data for mediation analyses of GLP-1RAs, including the LEADER [1, 3, 4] and other cardiovascular outcome trials [2, 4]. We therefore selected individual-level data from the LEADER [6] and SUSTAIN-6 [7] trials for the present analyses, which offered comprehensive follow-up schedules and prespecified biomarker assessments essential for temporal mediation analysis. The CONSORT flow diagrams for the study population and associated baseline characteristics tables can be found in the original published articles [6, 7]. To comply with the Declaration of Helsinki, both trials adhered to the policy of the institutional review board or ethics committee at the corresponding study site, with written informed consent provided to the trial participants at enrollment.

### Study outcome and mediator selection

The outcome of interest in this post-hoc analysis was 3P-MACE, namely nonfatal stroke, nonfatal myocardial infarction, and cardiovascular death, consistent with the primary outcome in the LEADER [6] and SUSTAIN-6 [7] trials. HbA_1c_, UACR, and SBP were selected as the mediators in this mediation analysis because they proved to be the most dominant mediators of liraglutide for 3P-MACE [1, 3].

### Causal mediation analysis

A continuous-time mediational g-formula proposed by Aalen et al. [8] (hereafter referred to as the Aalen method) was used to investigate the proportion of liraglutide- and semaglutide-associated effects on 3P-MACE that were mediated by HbA_1c_, UACR, and SBP over time. Compared to traditional mediation analyses using survival analyses (e.g., a Cox model), this approach relaxes the proportional hazard assumption (as one of the key assumptions of the Cox model) and allows a time-dependent consideration of mediators in survival analyses, thereby providing the dynamic impact of a given mediator over time [8]. In the Aalen method, a linear model was first used to describe the association of the exposure with a mediator. Additive hazard models were then utilized to separate and quantify the direct effect of exposure on the study outcome and the indirect effect of exposure through a mediator on the outcome over time. The sum of the direct and indirect effects is the total effect of exposure on the outcome, which is presented in terms of a cumulative hazard scale with a positive (negative) hazard indicating an increased (reduced) 3P-MACE risk with the use of GLP-1RAs (i.e., liraglutide or semaglutide) compared to the placebo. The percentage mediation was then calculated as the indirect effect divided by the total effect, and the corresponding confidence interval (CI) was estimated using 200 bootstrap replications [8]. This approach can occasionally yield negative values or upper confidence bounds exceeding 100% due to sampling variability, which should be interpreted as statistical uncertainty rather than literal mediation outside the 0–100% range. Supplementary Fig. 1 illustrates a step-by-step workflow of this study, with detailed model assumptions and equations provided in Supplementary Table 2. All HbA_1c_, UACR, and SBP values collected at the scheduled visits after treatment initiation (Supplementary Table 3) were utilized in this post-hoc analysis to assess the temporal mediation effects of these biomarkers. All analyses were based on observed data. In line with the trial protocols [6, 7], biomarker data were assumed to be missing at random across treatment groups, and no imputation was thus performed to avoid the introduction of bias. Since the last visits of trial participants for laboratory tests were at 36 and 24 months following treatment initiation in the LEADER [6] and SUSTAIN-6 [7] trials, respectively, the biomarker data available after these time points were not utilized in the analysis. Therefore, the first-, second-, and third-year percentage mediations and their 95% CIs are reported in the analysis using the LEADER [6] trial data, and only the first- and second-year percentage mediations are reported in the analysis using the SUSTAIN-6 [7] trial data. Moreover, subgroup analyses were performed to determine the heterogeneity in the mediation effects. As shown in Supplementary Table 4, in the LEADER [6] trial, the treatment effect of liraglutide on 3P-MACE was significantly modified by the patients’ baseline eGFR (i.e., *p*-value for interaction: 0.01) and cardiovascular risks (*p*-value for interaction: 0.04). Potential effect modifications also appeared in the SUSTAIN-6 [7] trial but did not reach statistical significance levels. The subgroups of patients with an eGFR < 60 ml/min/1.73 m^2^ or with an age of ≥ 50 years and established CVDs were thus analyzed in this study. All statistical analyses were performed using SAS software version 9.4 and R software. The R codes (“*analysis_simulation.R*”, “*my.additive.new.R*” and “*my.lm.alt.R*”) for implementing the Aalen method are available in the supplementary of the original paper by Aalen et al. [8].

## Results

In the LEADER trial, study populations aged between 64.2 (standard deviation: 7.2) and 64.4 (7.2) years old, with 64.0％–64.5% of male participants, and having 12.8 (8.0)–12.9 (8.1) years of diabetes duration, while in the SUSTAIN-6 trial, those were with age of 64.4 (7.5)–64.8 (7.6) years old, of a range of 58.5％–63.3% male subjects, and with diabetes duration of 13.2 (7.4)–14.3 (8.2) years. Of note, those established with CVDs (e.g., symptomatic coronary heart disease or asymptomatic cardiac ischemia, myocardial infarction, heart failure [New York Heart Association class II–III], ischemic, hemorrhage stroke, or transient ischemic attack) accounted for 14.0％–35.4% and 13.7％–61.9% in the LEADER and SUSTAIN-6 trials, respectively (Supplementary Table 5). Over a median follow-up period of 3.8 years in the LEADER trial, 608 and 694 3P-MACEs occurred in the liraglutide (*n* = 4668) and placebo (*n* = 4672) groups, respectively. In the SUSTAIN-6 trial, with a median follow-up of 2.1 years, 108 and 146 3P-MACE events occurred in the semaglutide (*n* = 1648) and placebo (*n* = 1649) groups, respectively. The proportion of patients lost to follow-up or withdrawn was low in both trials (0.3% in LEADER and 1.9% in SUSTAIN-6). Biomarker data were assumed to be missing at random across groups, and analyses were thus conducted using available data without imputation. As shown in Supplementary Table 4, GLP-1RA treatment with liraglutide or semaglutide significantly reduced the risk of 3P-MACE compared with placebo, with hazard ratios (95% CIs) of 0.87 (0.78–0.97) and 0.74 (0.58–0.95), respectively. As detailed in Table 1, the estimated total effects on the cumulative hazard scale varied slightly across the three mediators (HbA_1c_, UACR, and SBP), ranging from − 0.0176 to −0.0180 for liraglutide and from − 0.0734 to −0.1468 for semaglutide. Supplementary Fig. 2 also shows the dynamic pattern of direct, indirect, and total effects over time and the negative hazards throughout the trial periods. This variation reflects differences in data availability (i.e., the frequency of biomarker measurement) and model specification for each mediator. Importantly, all estimates consistently suggest risk reduction in 3P-MACE with liraglutide and semaglutide versus placebo. Notably, among the three mediators, HbA_1c_ contributed the most to liraglutide’s effects on 3P-MACE, accounting for 38.2% (95%CI 13.8％–194.5%), followed by UACR at 17.9% (8.5％–61.9%) and SBP at 6.8% (2.2％–31.0%). HbA_1c_ accounted for by 51.8% (29.6％–86.7%) of semaglutide’s effects on 3P-MACE, followed by UACR at 12.4% (6.1％–32.4%) and SBP at 6.7% (2.9％–11.7%) (Table 1).

**Table 1:** Total, indirect, and direct effects (in cumulative hazard scale) and percentage mediation of GLP-1RA treatments (i.e., liraglutide or semaglutide) versus placebo on 3P-MACE at end of trial period

	Total effect	Direct effect through treatment	Indirect effect through mediator	Percentage mediation, % (95% CI)
HbA_1c_ as mediator				
Liraglutide	−0.0177	−0.0109	−0.0067	38.2% (13.8％–194.5%)
Semaglutide	−0.1035	−0.0498	−0.0536	51.8% (29.6％–86.7%)
UACR as mediator				
Liraglutide	−0.0176	−0.0144	−0.0031	17.9% (8.5％–61.9%)
Semaglutide	−0.0734	−0.0643	−0.0091	12.4% (6.1％–32.4%)
SBP as mediator				
Liraglutide	−0.0180	−0.0168	−0.0012	6.8% (2.2％–31.0%)
Semaglutide	−0.1468	−0.1369	−0.0099	6.7% (2.9％–11.7%)

MACE, major adverse cardiovascular event; GLP-1RA, glucagon-like peptide-1 receptor agonist; CI, confidence interval; HbA_1c_, glycated hemoglobin; UACR, urine albumin-to-creatinine ratio; SBP, systolic blood pressure

**Fig. 1:**
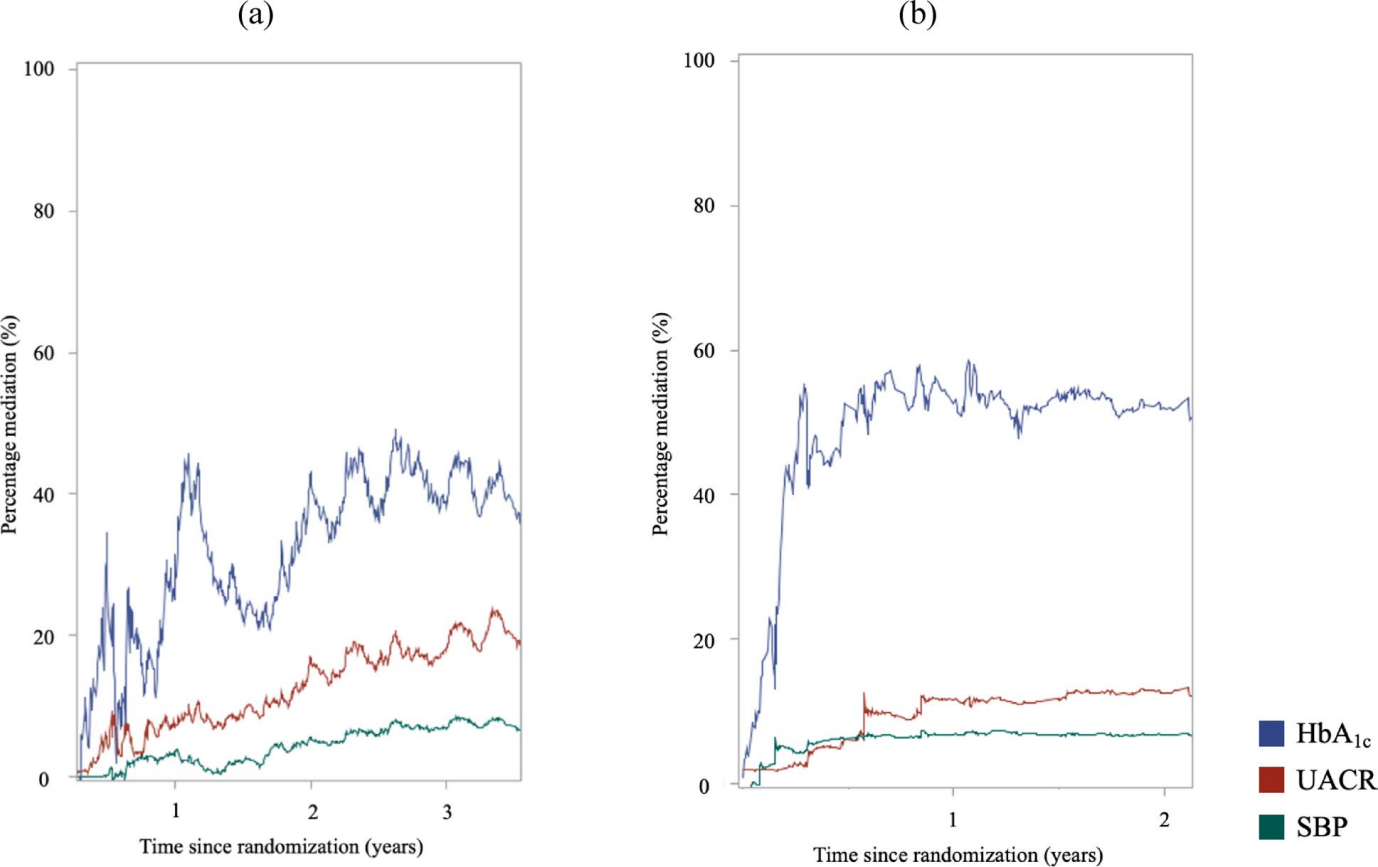
Results of Aalen method for percentage mediation of treatment effect on MACE outcome for HbA_1c_, UACR, and SBP over 3 years of trial period in (**a**) LEADER trial and (**b**) SUSTAIN-6 trial. Abbreviations: MACE, major adverse cardiovascular event; HbA_1c_, glycated hemoglobin; UACR, urine albumin-to-creatinine ratio; SBP, systolic blood pressure.

The percentage mediations of HbA_1c_, UACR, and SBP over time for liraglutide- and semaglutide-associated effects on 3P-MACE are presented in Table 2; Fig. 1. Over three years of follow-up, the contribution of HbA_1c_ to liraglutide’s effect was 31.3% in the first year, 42.9% in the second year (a relative change of + 37% compared with year 1), and 38.2% in the third year (+ 22% relative to year 1). UACR contributed 7.6%, 16.7% (+ 119%), and 17.9% (+ 136%) across the three years, while SBP contributed 3.2%, 5.6% (+ 75%), and 6.8% (+ 112%), respectively. For semaglutide, the percentage mediation values were 52.5% and 51.8% for HbA_1c_, 11.5% and 12.4% for UACR, and 6.8% and 6.7% for SBP across the first two years, showing minimal relative change.

**Table 2:** Results of Aalen method for percentage mediations (%) with corresponding 95% CIs of MACE with liraglutide versus placebo and semaglutide versus placebo for HbA_1c_, UACR, and SBP over 3 years (main analyses)

	Liraglutide			Semaglutide		
	HbA_1c_	UACR	SBP	HbA_1c_	UACR	SBP
1-year percentage mediation, %	31.3 (11.9–150.6)	7.6 (-60.7–83.9)	3.2 (-8.7–18.1)	52.5 (26.8–97.9)	11.5 (5.5–44.0)	6.8 (3.2–11.5)
2-year percentage mediation, %	42.9 (13.9–217.6)	16.7 (6.8–173.3)	5.6 (-0.4–87.0)	51.8 (29.6–86.7)	12.4 (6.1–32.4)	6.7 (2.9–11.7)
3-year percentage mediation, %	38.2 (13.8–194.5)	17.9 (8.5–61.9)	6.8 (2.2–31.0)	–	–	–

CI, confidence interval; MACE, major adverse cardiovascular event; HbA_1c_, glycated hemoglobin; UACR, urine albumin-to-creatinine ratio; SBP, systolic blood pressure

Heterogeneity in the contributions of mediation by HbA_1c_, UACR, and SBP to the effects of liraglutide and semaglutide on 3P-MACE across different patient subgroups was found. As shown in Table 3, in the third year, liraglutide’s effects were significantly mediated by UACR (11.8% [4.9％–30.2%]) and SBP (5.1% [1.7％–15.1%]) for patients with eGFR < 60 ml/min/1.73 m^2^, whereas HbA_1c_ (7.2％ [−8.1％ to 24.5％]) was not a significant mediator. In contrast, significant HbA_1c_-mediated effects (35.2% [8.5％–75.3%]) were observed in those receiving semaglutide, and UACR- and SBP-mediated effects were 11.2% (4.6％–22.1%) and 6.3% (3.0％–10.0%), respectively. For patients aged ≥ 50 years with established CVDs, the contributions of HbA_1c_, UACR, and SBP to the effects of liraglutide were 25.3% (9.0％–77.2%), 13.8% (5.9％–64.1%), and 5.4% (1.4％–14.4%), respectively, and those to the effects of semaglutide were 51.2% (32.1％–87.4%), 6.9% (1.9％–21.3%), and 7.2% (3.3％–13.0%), respectively. Details of percentage mediations over 3 years are given in Table 3.

**Table 3:** Results of Aalen method for percentage mediations with corresponding 95% CIs of MACE with liraglutide versus placebo and semaglutide versus placebo for HbA_1c_, UACR, and SBP across patient subgroups (subgroup analyses)

	Liraglutide			Semaglutide		
	HbA_1c_	UACR	SBP	HbA_1c_	UACR	SBP
Patients with eGFR < 60 ml/min/1.73 m^2^ (*n* = 2158 in LEADER trial; *n* = 939 in SUSTAIN-6 trial)						
1-year percentage mediation, %	9.5 (−114.2 to 276.9)	4.5 (−7.9 to 69.6)	2.2 (−11.8 to 37.4)	40.5 (2.9 to 151.0)	11.1 (0.6 to 75.8)	13.7 (3.0 to 87.7)
2-year percentage mediation, %	6.1 (−14.5 to 29.4)	8.0 (2.3 to 30.3)	5.6 (−0.2 to 26.0)	35.2 (8.5 to 75.3)	11.2 (4.6 to 22.1)	6.3 (3.0 to 10.0)
3-year percentage mediation, %	7.2 (−8.1 to 24.5)	11.8 (4.9 to 30.2)	5.1 (1.7 to 15.1)	–	–	–
Patients aged ≥ 50 years and with established CVDs (*n* = 7598 in LEADER trial; *n* = 2735 in SUSTAIN-6 trial)						
1-year percentage mediation, %	23.3 (−2.5 to 137.6)	7.5 (−21.5 to 73.1)	3.6 (−0.1 to 14.2)	50.4 (25.7 to 91.2)	7.7 (2.5 to 23.3)	7.7 (3.1 to 14.6)
2-year percentage mediation, %	32.3 (8.1 to 112.3)	13.2 (5.1 to 58.3)	4.9 (1.2 to 23.8)	51.2 (32.1 to 87.4)	6.9 (1.9 to 21.3)	7.2 (3.3 to 13.0)
3-year percentage mediation, %	25.3 (9.0 to 77.2)	13.8 (5.9 to 64.1)	5.4 (1.4 to 14.4)	–	–	–

Abbreviations: CI, confidence interval; MACE, major adverse cardiovascular event; HbA_1c_, glycated hemoglobin; UACR, urine albumin-to-creatinine ratio; SBP, systolic blood pressure; eGFR, estimate glomerular filtration rate; CVDs, cardiovascular diseases

## Discussion

This study shows that cardiovascular benefits associated with liraglutide and semaglutide are predominantly mediated through HbA_1c_, followed by UACR and SBP. However, a discrepancy in the temporal mediation patterns of these biomarkers over time between liraglutide and semaglutide on cardiovascular outcomes was found. Such mediation effects/patterns were generally consistent across the patient subgroups (e.g., those aged ≥ 50 years and with established CVDs), but not in those with impaired renal function. These findings underscore the importance of timely glycemic control and comprehensive diabetes management when prescribing GLP-1RA therapies for patients with T2D, in order to reduce the risk of cardiovascular complications.

### Discrepancy in temporal mediation patterns of HbA_1c_ on liraglutide- and semaglutide-associated cardiovascular benefits

Compared to UACR and SBP, HbA_1c_ predominantly contributed to GLP-1RA-associated cardiovascular benefits. It is reasonable that the indirect effect via HbA_1c_ accounts for a larger portion of GLP-1RAs’ (high-potency therapy [9]) total effect on 3P-MACE compared with its direct effect, given the well-established association between HbA_1c_ reduction and lower cardiovascular risk [10]. The estimated HbA_1c_-mediated liraglutide’s and semaglutide’s effects estimated from the current study fell within the ranges reported in previous studies (i.e., 9.9％–82.0% [1–3]). Differences across studies likely reflect variations in analytic methods (e.g., Cox-based vs. additive hazard mediation models), study populations, follow-up durations, and definitions of mediators and outcomes. Our use of the Aalen additive hazards approach provides complementary evidence, while acknowledging that direct comparisons with prior analyses should be made cautiously. The temporal mediation pattern of HbA_1c_ differed between liraglutide and semaglutide users. That is, for liraglutide users, the percentage mediation of HbA_1c_ increased slowly following treatment initiation and steadily increased with continuous use of liraglutide; in contrast, for semaglutide users, the HbA_1c_-mediated effects sharply increased at the beginning of treatment initiation and reached a plateau at around half a year of follow-up. Differences in glucose-lowering efficacy and essential pharmacokinetic features between liraglutide and semaglutide may explain such variation. First, as shown in Supplementary Fig. 3a, b, the magnitude of HbA_1c_ reduction by liraglutide was most significant at week 12 since treatment initiation. Afterward, HbA_1c_ in the liraglutide group gradually increased and got closer to that of the placebo group. In contrast, using semaglutide modestly reduced HbA_1c_ at week 8; HbA_1c_ reached its lowest level at week 16 and then remained constant until the end of the study follow-up. Hence, compared to the use of liraglutide, semaglutide could offer better glycemic control in T2D patients (mean difference in HbA_1c_ between groups at the end of the follow-up of the LEADER [6] and SUSTAIN-6 [7] trials: −0.4% and − 0.7 to 1.0%, respectively). Moreover, the pharmacokinetic properties of liraglutide and semaglutide, especially the half-lives (i.e., liraglutide versus semaglutide: approximately 13 h versus 7 days, allowing for once-daily/once-weekly administration) [11], also support the mediation variation by HbA_1c_. Given the long-acting GLP-1RAs that can maintain the effective drug concentration in plasma, semaglutide therapy provides sustained efficacy in glycemic control. The SUSTAIN-10 trial further reported supporting findings that compared to once-daily liraglutide, once-weekly semaglutide was superior in terms of HbA_1c_ reduction (i.e., −0.69%, 95% CI −0.82% to −0.56%) among T2D patients [12].

Compared to the sizeable mediation impact through HbA_1c_, UACR and SBP modestly contributed 12.4–17.9% and 6.7–6.8% of the mediation effects on liraglutide- and semaglutide-associated cardiovascular benefits, respectively. The temporal mediation patterns of these two mediators were generally comparable for liraglutide and semaglutide. The lower percentage mediation of UACR and SBP can be explained by differences between individuals and between groups. Specifically, although the UACR values following the use of liraglutide or semaglutide over the trial period were lower than those of patients receiving placebo (Supplementary Fig. 3c, d), the reductions of UACR within individuals did not reach the minimal clinically important difference, namely 30% of UACR reduction [13]. Similarly, the minimal clinically important difference of SBP, namely a decrease of 2 mmHg [14], was not achieved for liraglutide versus placebo (−1.2 mmHg) [6] and semaglutide 0.5 mg versus placebo (−1.3 mmHg) [7] yet achieved for semaglutide 1.0 mg versus placebo (−2.6 mmHg) at the end of the trial, and thus the percentage mediation of SBP for GLP-1RA-associated cardiovascular benefits remained constant at a low level. These results imply that the reduction of UACR and SBP might not be directly linked to or fully explain liraglutide- or semaglutide-associated cardiovascular benefits, thereby explaining the limited mediation percentage of these two mediators observed in the present study.

### Heterogeneity of mediation impact of HbA_1c_ between liraglutide and semaglutide in patients with poor renal function

Consistent with the observed temporal patterns of mediation impacts of HbA_1c_, UACR, and SBP in the overall study populations, those in the subset of patients aged ≥ 50 years and with established CVDs were generally similar between liraglutide and semaglutide users. Such consistency between the overall trial participants and this subset of patients is expected since the LEADER [6] and SUSTAIN-6 [7] trials primarily recruited T2D patients with established CVDs or high cardiovascular risks. However, a discrepancy of the temporal mediation pattern of HbA_1c_ between liraglutide and semaglutide users in the subset of patients with impaired renal function (i.e., eGFR < 60 ml/min/1.73 m^2^) was found. This might be linked to differences in the area under the plasma drug (i.e., liraglutide or semaglutide) concentration-time curve (AUC) across T2D patients with different levels of renal function. That is, compared to those with normal renal function, patients with severe renal dysfunction had a 30% lower AUC [15] with liraglutide but had a 22% higher AUC with semaglutide [7]. Such difference in drug’s AUC might affect the glucose-lowering effect of liraglutide and semaglutide, potentially resulting in heterogeneous mediation percentages of HbA_1c_ on 3P-MACE between the two drugs in this vulnerable patient subgroup.

The mediation analysis findings highlight clinical implications for personalized management for T2D patients requiring GLP-1RA treatments. Both liraglutide and semaglutide effectively reduce cardiovascular risks mainly through glucose-lowering effect, while using semaglutide yields a greater HbA_1c_ reduction than liraglutide. Therefore, liraglutide and semaglutide can be utilized for GLP-1RA-naive patients or those with established CVDs, while semaglutide may be preferred for those with poor glycemic control or impaired renal function to optimize treatment benefits for cardiovascular outcomes.

### Supporting biological mechanism of GLP-1RA therapy

Existing evidence on the biological mechanisms of GLP-1RA therapy could explain the findings of the present mediation analyses [16–21]. Besides pancreatic islet cells, glucagon-like peptide-1 receptors are found in the kidney and blood vessels. Beyond glucose-lowering effects, GLP-1RAs can thus reduce albuminuria and blood pressure [22, 23]. Since GLP-1RAs can directly act on the glucagon-like peptide-1 receptors of glomeruli and arterioles, corresponding signals in cells lead to increased levels of cyclic adenosine monophosphate and the activation of protein kinase A, thereby decreasing the oxidative stress, inflammation, and kidney hypoxia, and ultimately improving albuminuria [16–18]. By inhibiting Na/H exchanger 3 in proximal tubules, GLP-1RAs can induce natriuresis to lower sodium retention in blood vessels, leading to decreased blood pressure [19–21]. Hence, given these biological benefits of GLP-1RAs and the suboptimal examination rates of these biomarkers in current practice, especially UACR [24] and SBP [25], our findings suggest the importance of continuously monitoring HbA_1c_ and improving examinations of UACR and SBP control with GLP-1RA therapies to maximize their benefits on 3P-MACE.

### Limitations

Some limitations to this study should be acknowledged. First, due to the use of trial data, our results might have limited generalizability to diverse patient populations in real-world settings. Although the overall rates of missing data were low, we acknowledge that missingness may still introduce bias. Our findings should therefore be interpreted with this limitation in mind. Second, some subgroups (e.g., patients with an eGFR level of < 60 ml/min/1.73 m^2^) had small numbers of patients, leading to variations of mediation results, namely wide CIs of percentage mediation. Further research with large sample sizes of patients is warranted to explore significant and/or different mediators among patient subgroups. Due to our limited analytic access to trial data, mediation effects across all prespecified subgroups could not be assessed. Broader access to patient-level trial data will be necessary to enable a comprehensive assessment of mediation effects across all relevant subgroups. Third, given the nature of post-hoc analysis, the lack of statistical power or insufficiency in biomarker measurements to detect unplanned mediation effects may exist [26]. The present findings should therefore be interpreted with caution. Fourth, the inability to handle multiple mediators simultaneously and adjust time-dependent confounders in the Aalen method [8] should be acknowledged. Although trial data can mitigate some of these threats through randomization and pre-specified measurement schedules, time-dependent confounding (e.g., due to intercurrent therapies or non-adherence) cannot be fully ruled out. Therefore, results should be interpreted with this limitation in mind, and evaluation of data quality is recommended to assess whether the assumptions of the Aalen method [8] are reasonably met. Additionally, our analyses were restricted to the additive hazard scale of the Aalen method, and we did not perform sensitivity analyses on alternative absolute scales (e.g., restricted mean survival time), which should be considered when interpreting the mediation results. Fifth, other potential mediators outside of HbA_1c_, UACR, and SBP were not available in the LEADER [6] and SUSTAIN-6 [7] trials and thus not examined in this study. Future research is warranted if sufficient biomarker data are collected to comprehensively explore additional mediators. Sixth, although both the LEADER [6] and SUSTAIN-6 [7] trials recruited generally similar populations of T2D patients with CV risks or established CVDs, differences in their inclusion and exclusion criteria between trials should be noted (Supplementary Table 1). These differences may have contributed to residual discrepancies in baseline characteristics, and therefore, direct comparisons of study findings should be interpreted with caution. Lastly, we assessed composite cardiovascular outcomes (i.e., 3P-MACE), rather than their individual components, in our analyses. Although both liraglutide and semaglutide demonstrated significant efficacy on 3P-MACE [6, 7], their effects on most individual components were neutral [6, 7]. This limited the feasibility of conducting meaningful mediation analyses for single outcomes. Considering that the clinical course of cardiovascular death, myocardial infarction, and stroke might differ, future research with larger or pooled data sources is warranted to investigate potential mediators at the component level.

## Conclusion

This study characterized the time-varying mediation effects on liraglutide- and semaglutide-associated cardiovascular outcomes and described the heterogeneity across patient subgroups. The findings highlight HbA_1c_ as the dominant mediator, while UACR and SBP contributed smaller proportions. These descriptive patterns may inform future research on differential treatment effects, but should not be interpreted as prescriptive guidance for clinical decision-making.

## Supplementary Information

Below is the link to the electronic supplementary material.

Supplementary Material 1

## Data Availability

In accordance with the data sharing commitments of Novo Nordisk, de-identified individual participant data, the study protocol, and a redacted clinical study report from the LEADER and SUSTAIN-6 trials were made available to authorized researchers via a secure SAS data platform. Access was granted after submission and approval of a research proposal, in accordance with the Independent Review Board Charter governing Novo Nordisk data-sharing. Data integrity and completeness were ensured by conducting all analyses within the secure platform environment, which maintained trial-level consistency and prevented download of raw datasets. The R codes used for the mediation analyses were identical to those published with the original methodological paper by Aalen et al. (*Biom J.* 2020 May;62(3):532-549.) and are available in its supplementary materials.

## Abbreviations

3P-MACE: Three-point major adverse cardiovascular event
CKD: Chronic kidney disease
CVD: Cardiovascular disease
eGFR: Estimated glomerular filtration rate
GLP-1RA: Glucagon-like peptide-1 receptor agonist
HbA_1c_: Glycated hemoglobin
T2D: Type 2 diabetes
SBP: Systolic blood pressure
UACR: Urine albumin-to-creatinine ratio
