# Supplementary material for: Temporal and subgroup disparities in mediation effects on cardiovascular outcomes with liraglutide and semaglutide: a post-hoc analysis of LEADER and SUSTAIN-6 trials
Source: Cardiovasc Diabetol. 2025 Nov 24;24:465. doi: 10.1186/s12933-025-03007-w (PMC12751512; doi:10.1186/s12933-025-03007-w)
Supplement: Supplementary file 1 — Supplementary Material 1 [file 12933_2025_3007_MOESM1_ESM.docx]

**Online only supplement**

Supplementary Table 1. Key inclusion and exclusion criteria for the LEADER and SUSTAIN-6 trials

Supplementary Table 2. Overview of the Aalen method and associated equations

Supplementary Table 3. Pre-specified time points for biomarker measurements in LEADER and SUSTAIN-6 trials

Supplementary Table 4. Hazard ratios and their 95% CIs of MACE associated with use of GLP-1RAs (i.e., liraglutide or semaglutide) versus placebo

Supplementary Table 5. Baseline characteristics of study populations in the LEADER and SUSTAIN-6 trials

Supplementary Figure 1. Overview of the workflow for mediation analyses using the Aalen additive hazards method

Supplementary Figure 2. Time-varying direct, indirect, and total effects (in cumulative hazard scale) of (a) liraglutide versus placebo and (b) semaglutide versus placebo on MACE

Supplementary Figure 3. HbA_1c_ (a, b), UACR (c, d), and SBP (e, f) changes over trial (i.e., LEADER, SUSTAIN-6) period stratified by treatment status (i.e., liraglutide or semaglutide versus placebo)

Supplementary Table 1. Key inclusion and exclusion criteria for the LEADER and SUSTAIN-6 trials

|  | **LEADER^1^** | **SUSTAIN-6^2^** |
| --- | --- | --- |
| Inclusion criteria | 1. Men or women with type 2 diabetes mellitus. 2. Age ≥ 50 years at screening and at least one of the below criteria:    1. prior myocardial infarction    2. prior stroke or prior TIA    3. prior coronary, carotid or peripheral arterial revascularization    4. >50% stenosis on angiography or other imaging of coronary, carotid or lower extremity arteries    5. history of symptomatic coronary heart disease documented by positive exercise stress test or any cardiac imaging, or unstable angina with ECG changes    6. asymptomatic cardiac ischemia documented by positive nuclear imaging test or exercise test or dobutamine stress echo    7. chronic heart failure NYHA class II-III    8. chronic renal failure, defined as glomerular filtration rate < 60 mL/min/1.73m2 per MDRD or < 60 mL/min per Cockroft-Gault formula   OR  age ≥ 60 years at screening and meeting at least one of the below criteria:   1. microalbuminuria or proteinuria 2. hypertension and left ventricular hypertrophy by ECG or imaging 3. left ventricular systolic or diastolic dysfunction by imaging 4. ankle/brachial index <0.9. 5. Anti-diabetic drug naïve or treated with one or more oral anti-diabetic drugs or treated with human NPH insulin or long-acting insulin analogue, alone or in combination with OAD(s). 6. HbA_1c_ ≥ 7.0% at screening. | 1. Men and women with type 2 diabetes mellitus. 2. Age ≥50 years at screening and clinical evidence of cardiovascular disease or age ≥60 years at screening and subclinical evidence of cardiovascular disease. 3. Anti-diabetic drug naïve, or treated with one or two OAD(s), or treated with human NPH insulin or long-acting insulin analogue or pre-mixed insulin, both types of insulin either alone or in combination with one or two OAD(s). 4. HbA_1c_ ≥ 7.0% at screening. |
| Exclusion criteria | 1. Type 1 diabetes 2. Use of a GLP-1RA (exenatide, liraglutide or other) or pramlintide or any DPP-4i within the 3 months prior to screening 3. Use of insulin other than human NPH insulin or long-acting insulin analogue within 3 months prior to screening. Short-term use of other insulin during this period in connection with intercurrent illness is allowed, at Investigators discretion 4. Acute decompensation of glycemic control requiring immediate intensification of treatment to prevent acute complications of diabetes (e.g., diabetes ketoacidosis) in the previous 3 months 5. An acute coronary or cerebrovascular event in the previous 14 days 6. Currently planned coronary, carotid or peripheral artery revascularization 7. Chronic heart failure NYHA class IV 8. Current continuous renal replacement therapy 9. eGFR (as per MDRD) < 30 mL/min/1.73m2 at screening. The criterion is applicable after a target number of 220 subjects with eGFR < 30 mL/min are randomized 10. End stage liver disease, defined as the presence of acute or chronic liver disease and recent history of one or more of the following: ascites, encephalopathy, variceal bleeding, bilirubin ≥ 2.0 mg/dL, albumin level ≤ 3.5 g/dL, prothrombin time ≥ 4 seconds prolonged, international normalized ratio ≥1.7 or prior liver transplant 11. A prior solid organ transplant or awaiting solid organ transplant 12. Malignant neoplasm requiring chemotherapy, surgery, radiation or palliative therapy in the previous 5 years. Patients with intraepithelial squamous cell carcinoma of the skin (Bowen’s disease) treated with topical 5FU and subjects with basal cell skin cancer are allowed to enter the trial 13. Family or personal history of multiple endocrine neoplasia type 2 or familial medullary thyroid carcinoma 14. Personal history of non-familial medullary thyroid carcinoma 15. Any acute condition or exacerbation of chronic condition that would in the Investigator's opinion interfere with the initial trial visit schedule and procedures 16. Known or suspected hypersensitivity to trial product(s) or related products 17. Known use of non-prescribed narcotics or illicit drugs 18. Simultaneous participation in any other clinical trial of an investigational agent. Participation in a clinical trial with investigational stent(s) is allowed 19. Previous participation in this trial. Participation is defined as randomized 20. Females of childbearing potential who are pregnant, breast-feeding or intend to become pregnant or are not using adequate contraceptive methods (adequate contraceptive measures as required by local law or practice) 21. Receipt of any investigational medicinal product within 30 days prior to this trial. | 1. Type 1 diabetes mellitus. 2. Use of GLP-1RA (exenatide, liraglutide, or other) or pramlintide within 90 days prior to screening. 3. Use of any DPP-4i within 30 days prior to screening. 4. Treatment with insulin other than basal and pre-mixed insulin within 90 days prior to screening - except for short-term use in connection with intercurrent illness. 5. Acute decompensation of glycemic control requiring immediate intensification of treatment to prevent acute complications of diabetes (e.g., diabetes ketoacidosis) within 90 days prior to screening. 6. History of chronic pancreatitis or idiopathic acute pancreatitis. 7. An acute coronary or cerebrovascular event within the previous 14 days. 8. Currently planned coronary, carotid or peripheral artery revascularization. 9. Chronic heart failure NYHA class IV. 10. Personal or family history of multiple endocrine neoplasia type 2 or familial medullary thyroid carcinoma. 11. Personal history of non-familial medullary thyroid carcinoma. 12. Screening calcitonin ≥50 ng/L. |

Abbreviations: TIA, transient ischemic attack; OAD, oral anti-diabetic drug; NPH, Neutral Protamine Hagedorn; ECG, electrocardiography; NYHA, New York Heart Association; MDRD, modification of diet in renal disease; GLP-1RA, glucagon-like peptide-1 receptor agonist; DPP-4i, dipeptidyl pepetidase-4 inhibitor; eGFR, estimated glomerular filtration rate.

References:

1. N Engl J Med. 2016 Jul 28;375(4):311-22. doi: 10.1056/NEJMoa1603827.
2. N Engl J Med. 2016 Nov 10;375(19):1834-1844. doi: 10.1056/NEJMoa1607141.

Supplementary Table 2. Overview of the Aalen method and associated equations

| **Step 1: Specifying the relationship between the intervention, mediators, and outcomes in the Aalen method^1^** | |
| --- | --- |
|  | Assumptions:   1. No unmeasured confounders. Baseline covariates are able to control the confounding effects between intervention and mediator, and those between intervention and outcome. And, randomized controlled trial data was therefore utilized in this study to meet this assumption. 2. The treatment effects can be distinctly separated by the effects mediated by the mediators (*I*^m^), and the effects directly from intervention not through mediators (*I*^d^). Of note, both of *I*^m^ and *I*_d_ is independent of each other. The mediators of subjects survived at given time point are conditional on the earlier mediators, *I*^m^, and baseline covariates. 3. The survival probability after the specific timepoint is conditional on the earlier mediators, *I*^m^, *I*^d^, and baseline covariates. |
| Denotes: “*B*” (baseline covariates); “*I*” (Intervention); “*I^m^*“ (mediated effect of intervention); “*I^d^*“ (direct effect of intervention); “*M*” (mediators); “*Y*” (remain survived at different time points, time 1, 2…and so on); “*T*” (outcomes occurred after time 2). |  |
|  |  |
| **Step 2: Integrating a linear model with an additive hazard model using the Aalen method^1^** | |
| 1. A linear model (to address the association of the intervention and a mediator)   Equation 1: *M(t)* = *α_0_(t)* + *α_A_(t)I, where all coefficients are time dependent.* | |
| 1. An additive hazard model (to quantify the treatment effects [presented in hazard scale])   Equation 2: *λ(t∣H(t))* =β_0_*(t)* + *β_M_M(t)* + *β_I_I, where all coefficients are time-dependent.* | |
| 1. Substitute Equation (1) into Equation (2):   Equation 3: *λ(t∣H(t))* =β_0_*(t)* + *β_M_ (α_0_(t)* + *α_A_(t)I)* + *β_I_I*   - *λ(t∣H)* = *β_M_α_A_(t)I* + *β_I_I (The symbol of time is ignored to provide concise equation.)* | |
| Raw data shall be prepared as the long-form datasets. The R code entitled “*analysis_simulation.R*” and associated functions entitled “*my.additive.new.R*” and “*my.lm.alt.R*” used for the Aalen method are available in the supplementary of the original paper^1^ by Aalen et al. | |
|  | |
| **Step 3: Estimating the percentage mediation and interpreting the results** | |
| 1. Indirect effect = *β_M_α_A_* 2. Direct effect *= β_I_* 3. Total effect =*β_M_α_A_* +*β_I_* 4. Percentage mediation = (*β_M_α_A_* / *β_M_α_A_* + *β_I_*)*100%   Of note, 200 bootstrap resampling is recommended to generate the 95% confidence intervals in the original paper^1^. | |

Reference:

1. Biom J. 2020 May;62(3):532-549. doi: 10.1002/bimj.201800263.

Supplementary Table 3. Pre-specified time points for biomarker measurements in LEADER and SUSTAIN-6 trials

| Weeks^*^ | 0 | 2 | 4 | 8 | 12 | 16 | 24 | 30 | 44 | 48 | 56 | 68 | 72 | 80 | 92 | 96 | 104 | 120 | 144 | 168 | 192 | 216 |
| --- | --- | --- | --- | --- | --- | --- | --- | --- | --- | --- | --- | --- | --- | --- | --- | --- | --- | --- | --- | --- | --- | --- |
| LEADER^1^ | | | | | | | | | | | | | | | | | | | | | | |
| HbA_1c_ | X |  |  |  | X |  | X |  |  | X |  |  | X |  |  | X |  | X | X | X | X | X |
| UACR | X |  |  |  |  |  |  |  |  | X |  |  |  |  |  | X |  |  | X |  | X |  |
| SBP | X |  |  |  |  |  | X |  |  | X |  |  |  |  |  | X |  |  | X |  | X |  |
| SUSTAIN-6^2^ | | | | | | | | | | | | | | | | | | | | | | |
| HbA_1c_ | X |  |  | X |  | X |  | X | X |  | X | X |  | X | X |  | X |  |  |  |  |  |
| UACR | X |  |  |  |  | X |  | X | X |  | X |  |  | X |  |  | X |  |  |  |  |  |
| SBP | X | X | X | X |  | X |  | X | X |  | X | X |  | X | X |  | X |  |  |  |  |  |

Abbreviations: UACR, urine albumin-to-creatinine ratio; SBP, systolic blood pressure.

*Week 0 indicates the time point for treatment randomization, whereas weeks 2-216 indicate the periods when the study subjects were under the treatment. “X” refers to the given biomarker being tested/measured.

References:

1. N Engl J Med. 2016 Jul 28;375(4):311-22. doi: 10.1056/NEJMoa1603827.
2. N Engl J Med. 2016 Nov 10;375(19):1834-1844. doi: 10.1056/NEJMoa1607141.

Supplementary Table 4. Hazard ratios and their 95% CIs of MACE associated with use of GLP-1RAs (i.e., liraglutide or semaglutide) versus placebo

|  | Liraglutide | | Semaglutide | |
| --- | --- | --- | --- | --- |
|  | HR (95% CI) | *p*-value for interaction | HR (95% CI) | *p*-value for interaction |
| **Total study populations** | 0.87 (0.78-0.97) |  | 0.74 (0.58-0.95) |  |
| **Renal function** |  |  |  |  |
| eGFR < 60 ml/min/1.73 m^2^ | 0.69 (0.57-0.85) | 0.01 | 0.84 (0.57-1.25) | 0.37 |
| eGFR ≥ 60 ml/min/1.73 m^2^ | 0.94 (0.83-1.07) |  | 0.67 (0.48-0.92) |  |
| **Established CVD** |  |  |  |  |
| age ≥ 50 years and with established CVD | 0.83 (0.74-0.93) | 0.04 | 0.72 (0.55-0.93) | 0.49 |
| age ≥ 60 years and with CV risks | 1.20 (0.86-1.67) |  | 1.00 (0.41-2.46) |  |

Abbreviations: HR, hazard ratio; CI, confidence interval; eGFR, estimated glomerular filtration rate; CVD, cardiovascular disease; CV, cardiovascular.

Supplementary Table 5. Baseline characteristics of study populations in the LEADER and SUSTAIN-6 trials

|  | **LEADER^1^** | | **SUSTAIN-6^2^** | | | |
| --- | --- | --- | --- | --- | --- | --- |
|  | Liraglutide  (n=4,668) | Placebo  (n=4,672) | Semaglutide 0.5 mg  (n=826) | Semaglutide 1.0 mg  (n=822) | Placebo 0.5 mg  (n=824) | Placebo 1.0 mg  (n=825) |
| Age, years (SD) | 64.2 (7.2) | 64.4 (7.2) | 64.6 (7.3) | 64.7 (7.1) | 64.8 (7.6) | 64.4 (7.5) |
| Male (%) | 64.5% | 64.0% | 59.9% | 63.0% | 58.5% | 61.5% |
| Diabetes duration, years (SD) | 12.8 (8.0) | 12.9 (8.1) | 14.3 (8.2) | 14.1 (8.2) | 14.0 (8.5) | 13.2 (7.4) |
| History of CVD (%)* | | | | | | |
| Symptomatic CHD or asymptomatic cardiac ischemia | 35.4% | 35.0% | 59.7% | 60.2% | 61.9% | 60.1% |
| Myocardial infarction | 31.4% | 30.0% | 32.2% | 32.1% | 32.4% | 33.3% |
| Heart failure (NYHA class II-III) | 14.0% | 14.0% | 24.3% | 21.9% | 23.1% | 25.0% |
| Ischemic, hemorrhage stroke or transient ischemic attack | 15.6% | 16.6% | 14.2% | 13.7% | 15.0% | 16.7% |
| Renal function (%) | | | | | | |
| eGFR < 60 ml/min/1.73 m^2^ | 23.9% | 22.3% | 30.2% | 26.7% | 29.5% | 27.5% |
| eGFR ≥ 60 ml/min/1.73 m^2^ | 76.1% | 77.7% | 69.7% | 73.3% | 70.5% | 72.4% |

Abbreviations: SD, standard deviation; CVD, cardiovascular disease; CHD, coronary heart disease; NYHA, New York Heart Association; eGFR, estimated glomerular filtration rate.

*Details of CVD breakdown are available in the main and supplementary tables of the original trials.

References:

1. N Engl J Med. 2016 Jul 28;375(4):311-22. doi: 10.1056/NEJMoa1603827.
2. N Engl J Med. 2016 Nov 10;375(19):1834-1844. doi: 10.1056/NEJMoa1607141.

Supplementary Figure 1. Overview of the workflow for mediation analyses using the Aalen additive hazards method

Legend:

Randomized treatment assignment (liraglutide or semaglutide vs placebo) was modeled to affect cardiovascular outcomes (3P-MACE) through both direct effects (*I^d^*) and indirect effects (*I^m^*) mediated by repeated biomarker measurements (HbA_1c_, UACR, SBP) collected at prespecified time points. The Aalen method integrates a linear model for the association between treatment and mediators with an additive hazards model for the outcome, allowing the total effect to be decomposed into direct and indirect components. The proportion mediated was calculated as indirect effect ÷ total effect, with 95% confidence intervals estimated using 200 bootstrap replications, as recommended in the original methodological paper by Aalen et al.

Supplementary Figure 2. Time-varying direct, indirect, and total effects (in cumulative hazard scale) of (a) liraglutide versus placebo and (b) semaglutide versus placebo on MACE

| Direct effect | Indirect effect | | | | | Total effect |
| --- | --- | --- | --- | --- | --- | --- |
| (a) | | | | | | |
| *HbA_1c_ as mediator* | | | | | | |
| 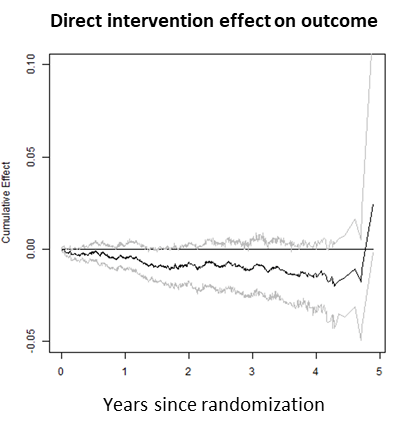 | 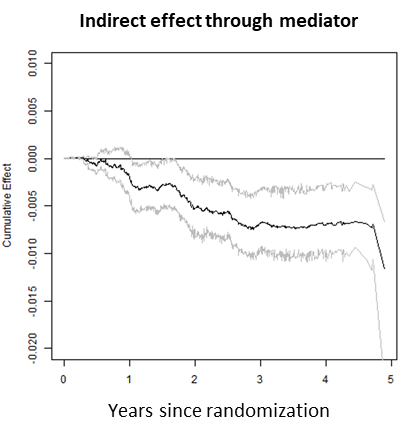 | | | | | 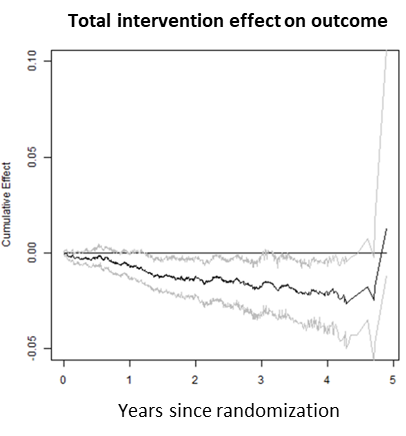 |
| *UACR as mediator* | | | | | | |
| 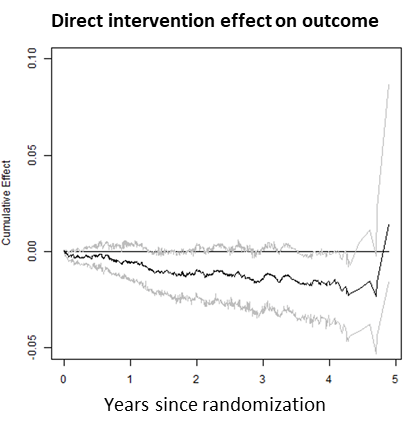 | 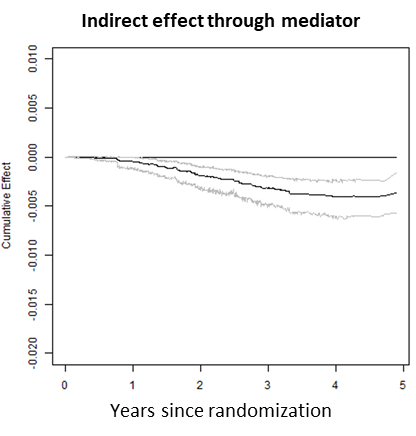 | | | | | 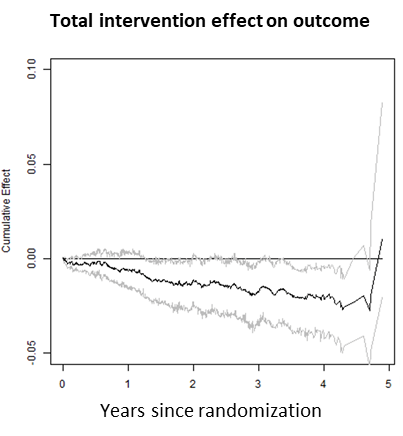 |
| *SBP as mediator* | | | | | | |
| 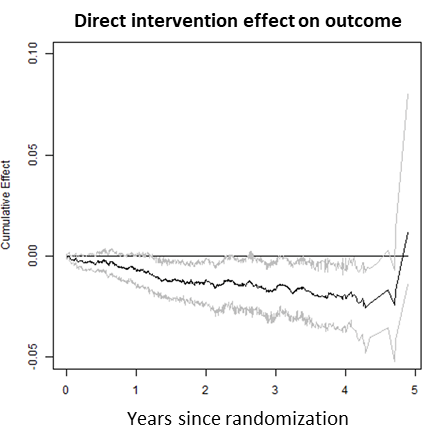 | 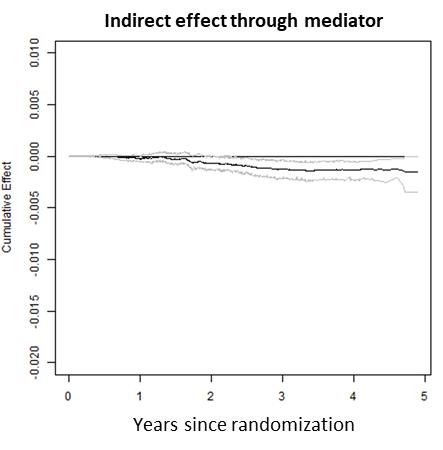 | | | | | 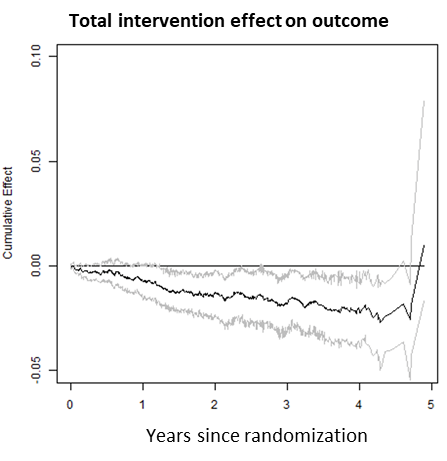 |
| (b) | | | | | | |
| *HbA_1c_ as mediator* | | | | | | |
| *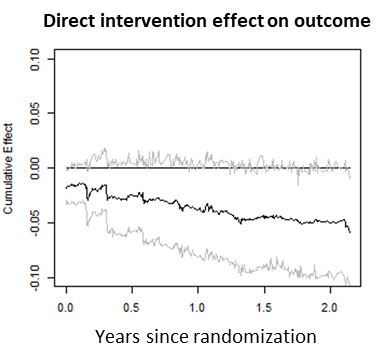* | 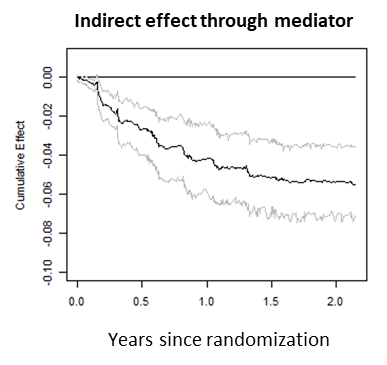 | | | | | 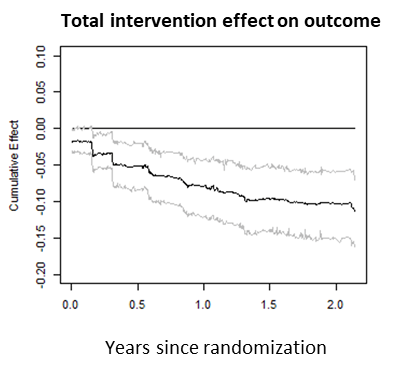 |
| *UACR as mediator* | | | | | | |
| *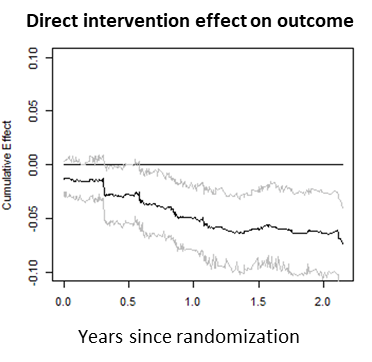* | | *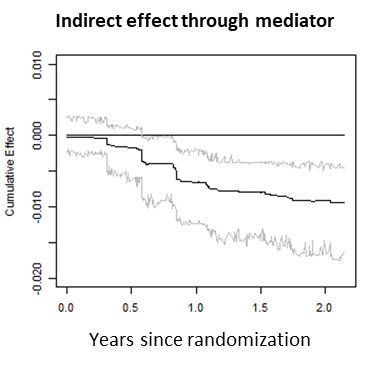* | | | 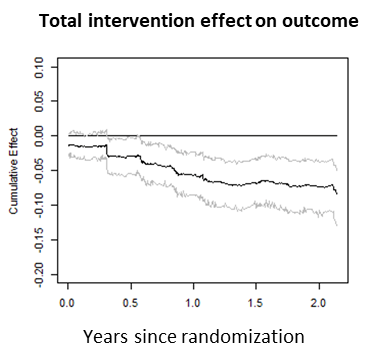 | |
| *SBP as mediator* | | | | | | |
| 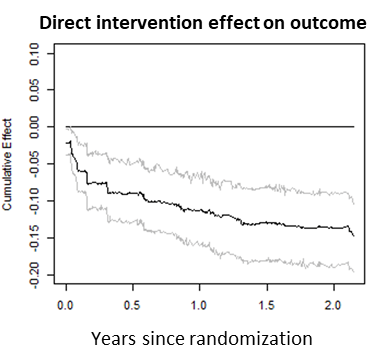 | | | 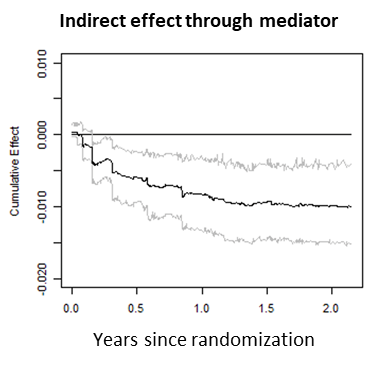 | 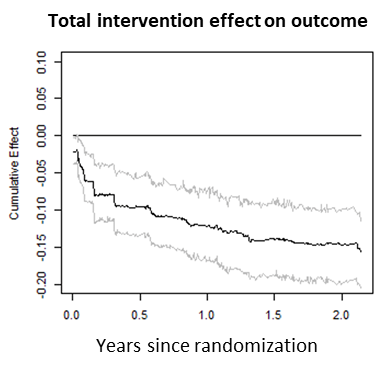 | | |

Abbreviations: HbA_1c_, glycated hemoglobin; UACR, urine albumin-to-creatinine ratio; SBP, systolic blood pressure.

Supplementary Figure 3. HbA_1c_ (a, b), UACR (c, d), and SBP (e, f) changes over trial (i.e., LEADER, SUSTAIN-6) period stratified by treatment status (i.e., liraglutide or semaglutide versus placebo)

| **LEADER trial** | **SUSTAIN-6 trial** |
| --- | --- |
|  |  |
| 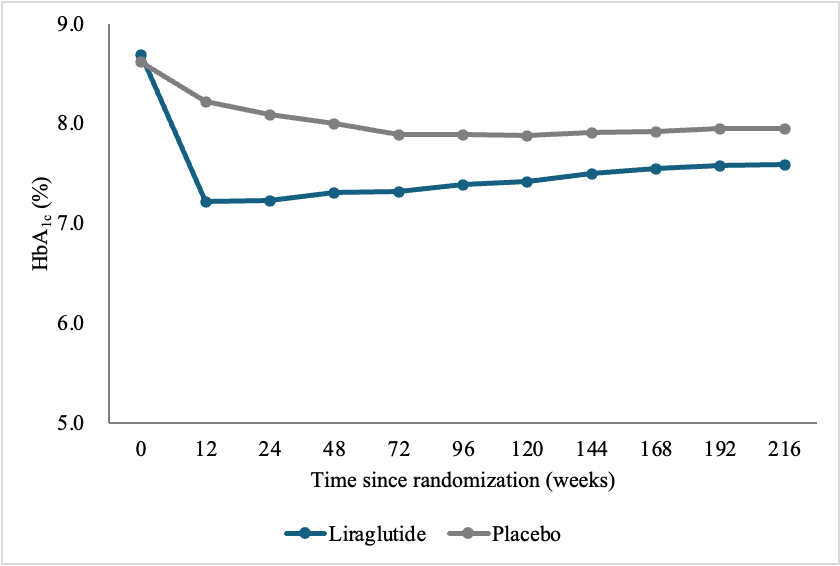 | 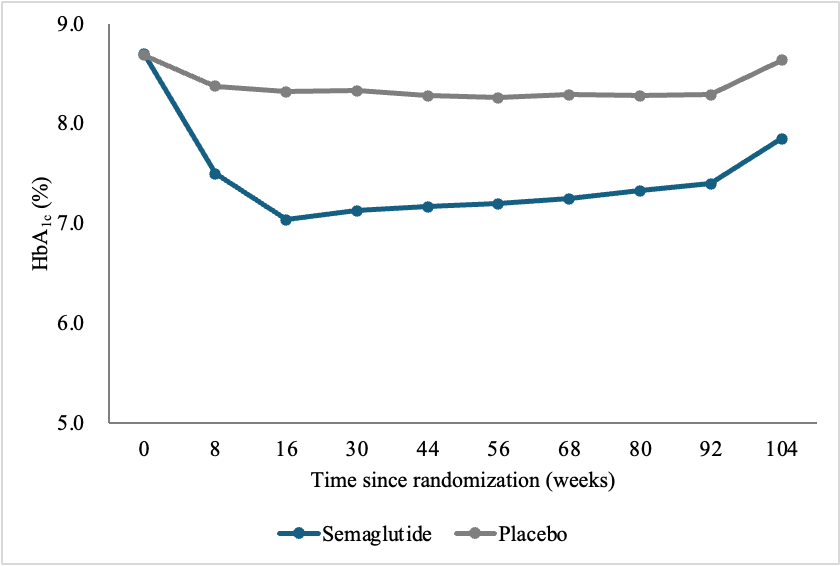 |
|  |  |
| 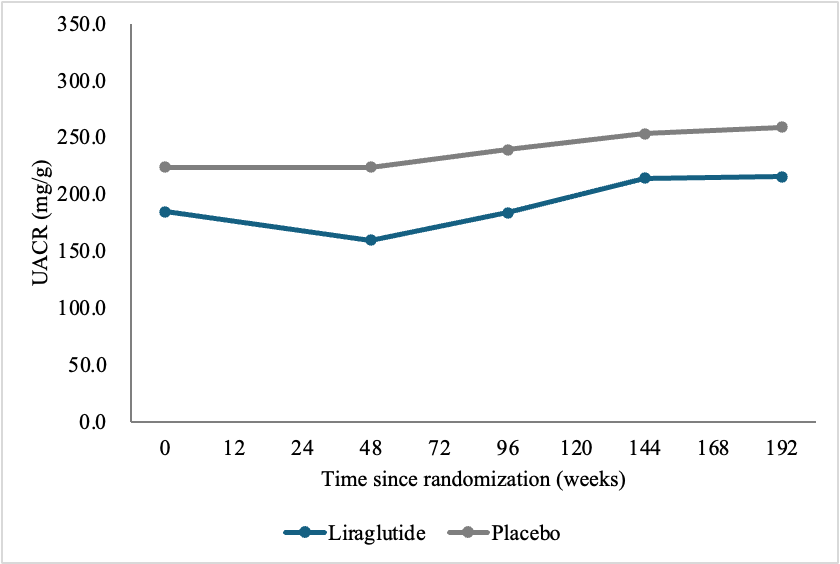 | 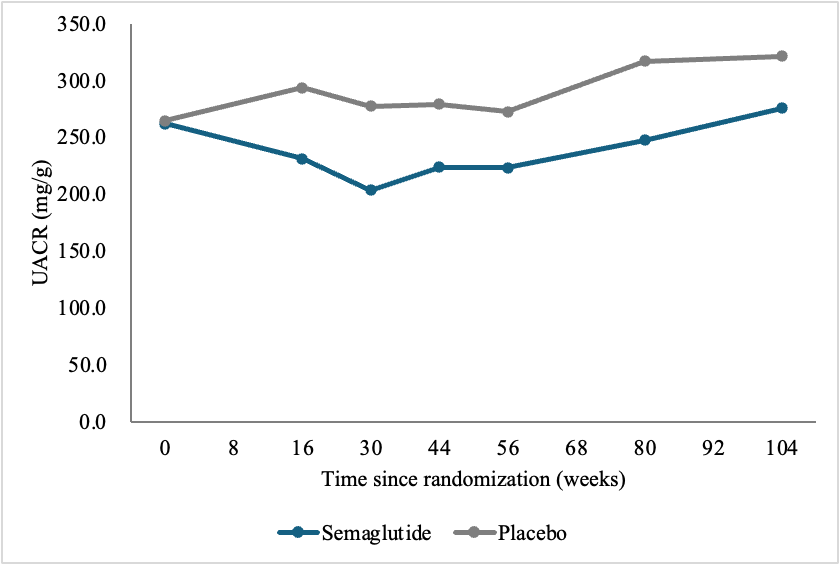 |
|  |  |
| 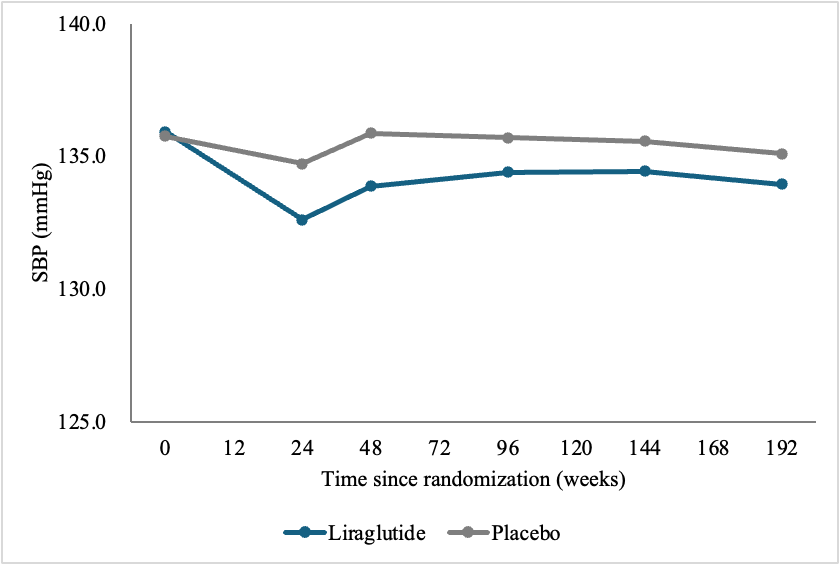 | 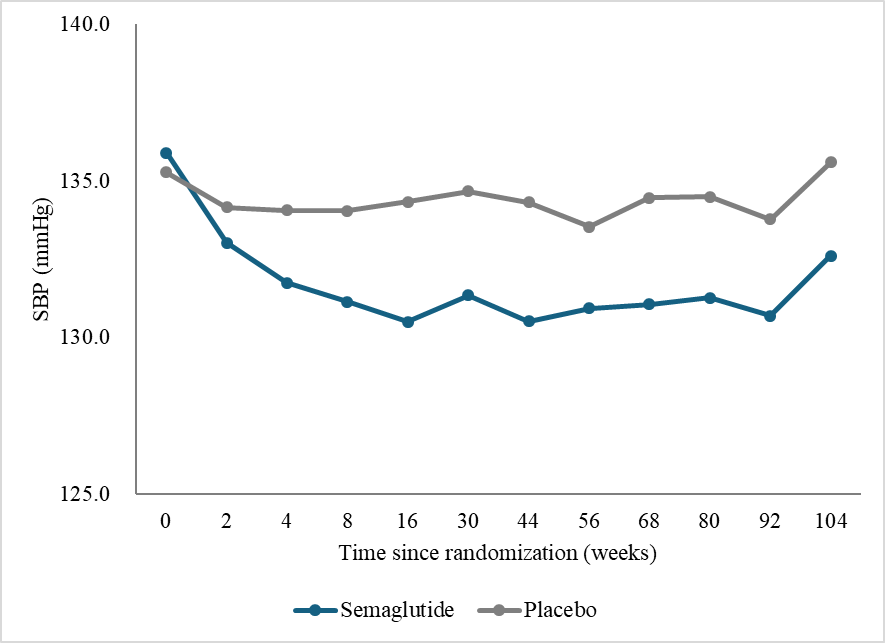 |

Abbreviations: HbA_1c_, glycated hemoglobin; UACR, urine albumin-to-creatinine ratio; SBP, systolic blood pressure.
